# Plugin Framework-Based Neuro-Symbolic Grounded Task Planning for Multi-Agent System

**DOI:** 10.3390/s21237896

**Published:** 2021-11-26

**Authors:** Jiyoun Moon

**Affiliations:** Department of Electronics Engineering, Chosun University, Gwangju 61452, Korea; jymoon@chosun.ac.kr

**Keywords:** neuro-symbolic, task planning, planning domain definition language, multi agent reinforcement learning, cooperative–competitive teaming

## Abstract

As the roles of robots continue to expand in general, there is an increasing demand for research on automated task planning for a multi-agent system that can independently execute tasks in a wide and dynamic environment. This study introduces a plugin framework in which multiple robots can be involved in task planning in a broad range of areas by combining symbolic and connectionist approaches. The symbolic approach for understanding and learning human knowledge is useful for task planning in a wide and static environment. The network-based connectionist approach has the advantage of being able to respond to an ever-changing dynamic environment. A planning domain definition language-based planning algorithm, which is a symbolic approach, and the cooperative–competitive reinforcement learning algorithm, which is a connectionist approach, were utilized in this study. The proposed architecture is verified through a simulation. It is also verified through an experiment using 10 unmanned surface vehicles that the given tasks were successfully executed in a wide and dynamic environment.

## 1. Introduction

The demands for robots are consistently increasing in various domains including ground, air, and the ocean as the robots are proven to be efficient based on the convergence of new technologies such as artificial intelligence and big data [1]. Unlike conventional robots displaying simple and repetitive movements and working only within a specific area, the robots developed in recent years are becoming more complicated as they are capable of performing in broader work areas [2]. There is a growing interest in a multi-agent system that can quickly execute tasks by efficiently dividing work into a broad range of areas compared to a single-agent system [3]. Some of the fields that utilize multi-agent systems include surveillance [4,5,6], data collection [7,8], and inspection [9,10,11]. Wang et al. [12] proposed a multi-agent system based on generalized covariance intersection for multi-view surveillance in centralized and decentralized situations. Laport et al. [13] proposed a multi-agent architecture for collecting massive data using mobile sensing devices. Jing et al. [14] introduced a coverage path planning framework for the large and complex structure inspection of multiple unmanned aerial vehicles.

Various studies on algorithms are being conducted on simultaneous localization and mapping (SLAM) [15,16,17], collision avoidance [18,19,20], and formation [21,22] for multi-agent systems. Jang et al. [23] introduced a collaborative monocular SLAM using the rendezvous generated as multiple robots execute tasks. Douthwaite et al. [24] analyzed several velocity-based multi-agent collision avoidance algorithms. Li and Zhou [25] proposed the slight-weight convolutional neural network as an end-to-end training method that can be applied to the visual scene recognition of a multi-agent system. Yan et al. [26] suggested a distributed adaptive event-triggered formation control strategy for the formation control of nonlinear heterogeneous agents and verified formation robustness when executing tracking and patrolling tasks. Accordingly, an array of research has been conducted recently on the operation of multi-agent systems. However, research on automated planning [27] for multi-agent systems, which is essential to high-level decision making, receives relatively little attention in these studies.

A large number of automated planning systems plan the sequence of actions for executing a given task using a planner [28,29,30,31] through symbolic representations of an environment. Such a symbolic approach represents knowledge with symbols and makes an inference system using rules and operations [32]. Galindo et al. [33] enabled robots to perform task planning based on higher levels of autonomy and intelligence using semantic knowledge. Wurm et al. [34] suggested a solution for solving the target assignment problem of heterogeneous agents based on temporal symbolic planning. Vasilopoulos et al. [35] proposed a planner that can execute tasks in partially known environments to provide a successful solution for the warehouseman’s problem [36]. The greatest advantage of symbolic task planning is that large sets of states can be inferred through symbolic-type partial state description and reasoning [37]. However, the methods that approach task planning problems through symbolic representations of an environment have the limitation of being unable to solve dynamic tasks efficiently in environments that change quickly [38].

Reinforcement learning, which is a learning process that achieves a given goal by interacting with an ever-changing environment in action, state, and reward information formats, is advancing quickly in the robotics field [39]. In particular, a great number of researchers are focusing on reinforcement learning methods for complicated multi-agent systems rather than a single robot [40,41,42]. Wang et al. [43] proposed the role-oriented multi-agent reinforcement learning (ROMA) framework in which each agent quickly learns based on their own pre-defined roles; Deka et al. [44] proposed a reinforcement learning method in a mixed cooperation–competition method. Lowe et al. [45] introduced a robust multi-agent policy training regimen based on an actor–critic method. Perrusquía et al. [46] proposed intelligent learning methods for the kinematic problem of redundant robots. Reinforcement learning, which is a network-based connectionist approach, has the advantage of being able to promptly respond to sudden changes but the disadvantage of a long learning time for planning the actions of an agent in a wide space.

**Figure 1:**
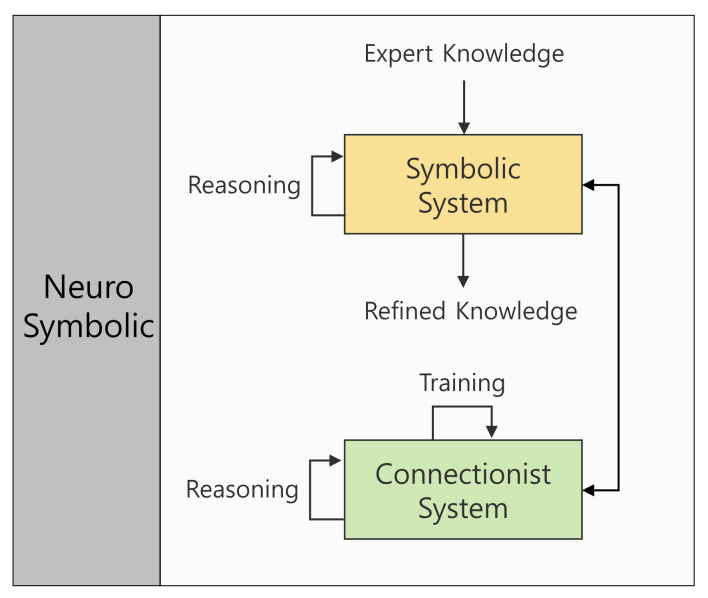
Neuro-symbolic approach: Connecting the symbolic system which outputs refined knowledge by reasoning the expert knowledge input in a symbolic representation format with the connectionist system which trains.

Symbolic and connectionist approaches, which are two well-known approaches in autonomous decision making, are collectively called neuro-symbolic and have been developed in the direction of becoming unified [47] while being applied to various automated planning systems by complementing each other’s strengths and weaknesses. The concept of a neuro-symbolic approach is as shown in Figure 1. A symbolic system outputs refined knowledge through reasoning based on expert knowledge as input. The connectionist system proceeds with training through reasoning. The system with an inter-connection between the symbolic system and the connectionist system is called a framework for neuro-symbolic integration. Umili et al. [38] suggested a model which learns symbolic representation from a continuous state space through deep reinforcement learning. Grounds and Kudenko [48] introduced the PLANQ-learning method, where the Q-learner and STRIPS planner are combined. Yang et al. [49] proposed the planning execution observation reinforcement learning (PEORL) framework in which symbolic planning is integrated with hierarchical reinforcement learning. Inala et al. [50] proposed employing neuro-symbolic transformers to solve the cooperative multi-agent communication problem. Kimura et al. [51] used the logical neural network to solve problems associated with text-based games. However, the majority of neuro-symbolic methods do not consider task planning methods in dynamic and wide areas for multi-agent systems. This study thus proposes a neuro-symbolic-based plugin framework for the intelligent task planning of multiple autonomous agents. The proposed architecture mitigates the limitations of the symbolic approach, which is vulnerable in a dynamic environment, by applying a network-based approach which is capable of a prompt response. Moreover, automated planning was enabled by reinforcement learning in a wide area through the planning domain definition language (PDDL) planner.

This paper is organized as follows. In Section 2, the neuro-symbolic-based plugin framework for the task planning of a multi-agent system is explained. In Section 3, the proposed architecture is verified through a simulation based on a competitive–cooperative scenario using 10 unmanned surface vehicles (USV). In Section 4, the significance of this study, which connects symbolic and connectionist approaches, is discussed. Lastly, the conclusion is presented in Section 5.

## 2. Methods

This section describes the neuro-symbolic framework for the task planning of a multi-agent system that is robust to a wide and dynamic area. The proposed architecture is explained by largely dividing it into symbolic planning, cooperative–competitive reinforcement learning, and a plugin framework for a neuro-symbolic approach. The details of the proposed architecture are as follows.

### 2.1. Symbolic Planning

**Figure 2:**
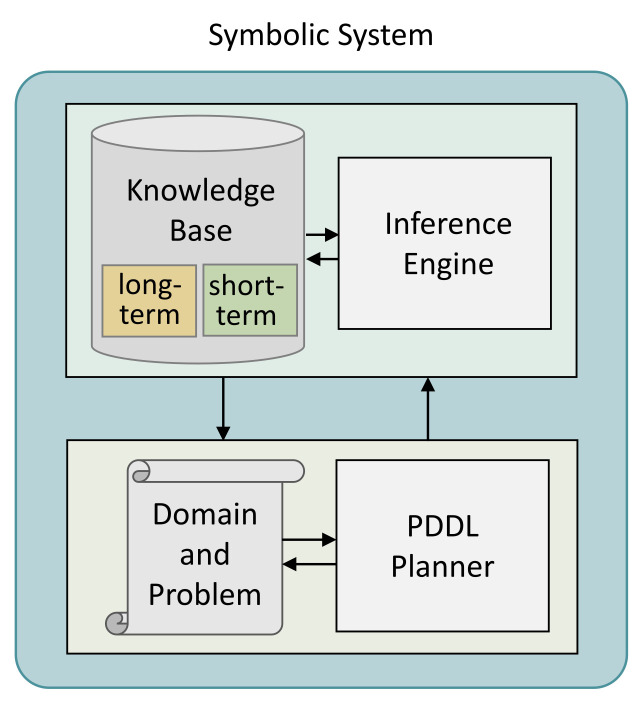
PDDL-based symbolic system, consisting of two parts: the first part is the knowledge base and interface engine, in which the domain information of task planning expressed with symbolic representation is saved; the second part is the PDDL-based domain and problem description and the PDDL planner for executing tasks by planning the sequence of actions.

The symbolic system of the proposed neuro-symbolic framework follows the ROSPlan [52], which is a robot operating system (ROS)-based automated planning method. The symbolic system designed in this study is shown in Figure 2. The knowledge base stores all of the long-term and short-term data. Long-term data comprise environment information expressed with symbols or agent information including performance, possible movements, and roles of different multi-agents. Short-term data comprise information acquired in real-time during task planning. In the interface engine, only the information required for executing given tasks is extracted from the knowledge base. The extracted information describes the PDDL domain and problem and generates actions of agents through a planner. Unlike conventional methods, the acquired information of multi-agents can be merged and new information can be inferred through reasoning by using the knowledge base in planning. A new PDDL problem can be automatically generated when re-planning is needed while executing tasks, since the changed information is updated in the knowledge base. Thus, the success rate of task execution can be increased.

**Figure 3:**
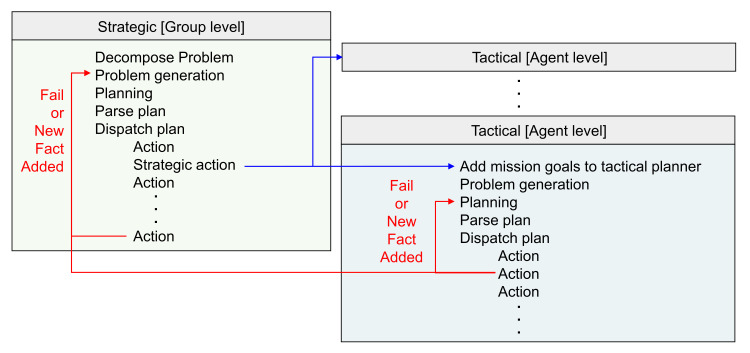
The flow of strategic and tactical planning: A plan is generated by dividing it into strategic and tactical levels for the task planning of a multi-agent system in a large area. At the strategic level, the plan is generated at the level of groups constituting the multi-agent system. At the tactical level, the sequence of actions of agents constituting each group is planned.

Strategic–tactical planning [53] is used for generating the sequence of actions of multi-agents in a wide area. The multi-agent system in this study consists of a central operating system (COP) which oversees the entire system by dividing the agents into groups for the operation. Groups are controlled at the strategic level, while each agent is controlled at the tactical level. The COP and the main agent of each group have a planner for hierarchical level planning. Therefore, strategic-level planning is executed in the COP planner, while tactical planning is executed in the planner of the main agent of each group. The overall process is as shown in Figure 3. First, the entire mission is divided into groups at the strategic level, problems are generated, and then a plan is generated using the COP planner. Subsequently, the generated plan is parsed, and then action and strategic action are dispatched to each group. During strategic action, the actions of all agents belonging to each group are planned in the planner at the tactical level, and the results are parsed and dispatched.

**Figure 4:**
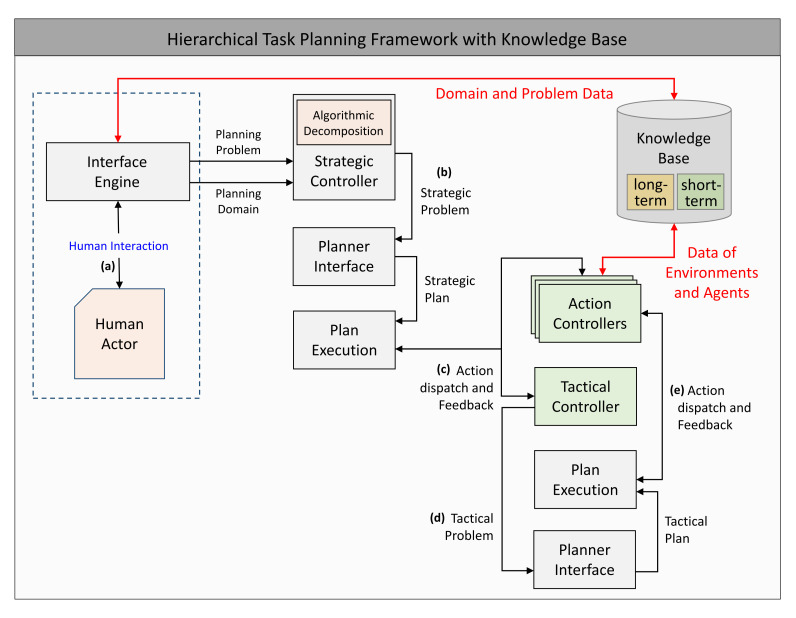
Hierarchical task planning framework with a knowledge base: The strategic level and tactical level each consist of the controller, planner interface, and plan execution. The environment and agent data required for planning are saved in the knowledge base, based on which the PDDL domain and problem are automatically generated in the interface engine.

**Figure 5:**
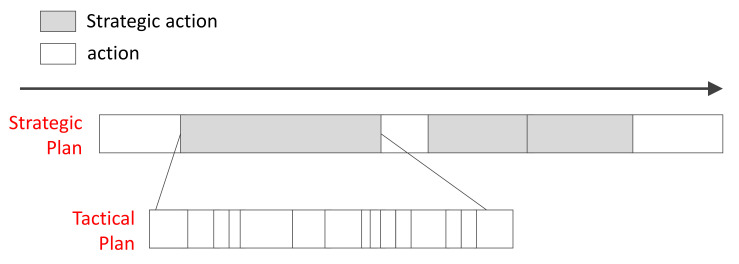
Strategic plan and tactical plan: The strategic plan consists of strategic action and action. The tactical plan is generated when a strategic action is performed and is executed.

The hierarchical framework for strategic–tactical planning shown in Figure 3 is as shown in Figure 4. Using domain-dependent information saved in the knowledge base and task goals given by a human actor, the PDDL domain and problems are automatically generated in the interface engine to be approved in a strategic controller. Algorithmic decomposition for the problem allocation of a multi-agent system at a group level is executed in the strategic controller, and the results are delivered as a planner interface and plan execution. The plan execution process at the strategic level is as shown in Figure 5. A strategic plan consists of an action and strategic action, while a strategic action consists of actions at the tactical level. In the tactical controller, a plan is generated by receiving the strategic action and is executed through the action controller.

Environment information and the sensor information of agents required while the plan is executed are updated in the knowledge base. As shown by the flow in the red arrows in Figure 3, planning is still possible based on the agents’ state or current situation updated in the knowledge base when an unexpected failure occurs or a new fact is added. Through the hierarchical task planning framework which includes the knowledge base, an efficient plan of a multi-agent system in a wide area is generated.

### 2.2. Cooperative–Competitive Reinforcement Learning

**Figure 6:**
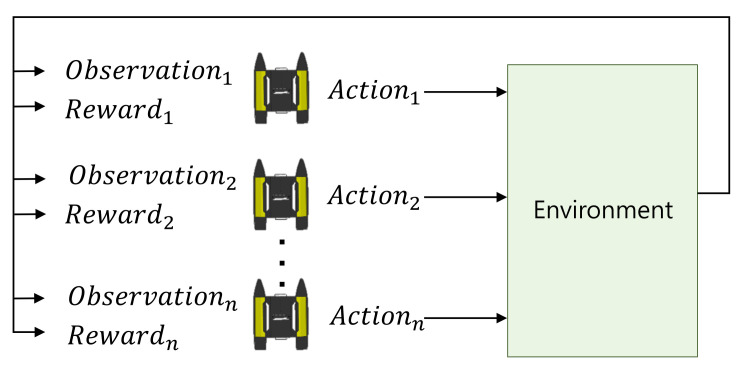
Multi-agent reinforcement learning: Multiple agents interact through observation and reward from the environment and learn the actions.

The essence of deep reinforcement learning is that agents interact in operating environments and the sequential decision-making problem is solved through a trial and error approach. Based on the observation from the environment and consistent interactions through a reward as shown in Figure 6, the policy is improved to maximize the gain of the reward. In most multi-agent reinforcement learning (MARL) systems, the policy of each agent is learned through mutual cooperation or competition between multiple agents, where all other agents are considered as an environment from the perspective of one agent. Consequently, each agent is operated independently. However, since a multi-agent system needs to behave organically in order to achieve a certain goal, a graph neural network (GNN)-based reinforcement learning approach [44] and multi-agent deep deterministic policy gradient (MADDPG) [45] approach for simultaneously training multiple agents are used.

**Figure 7:**
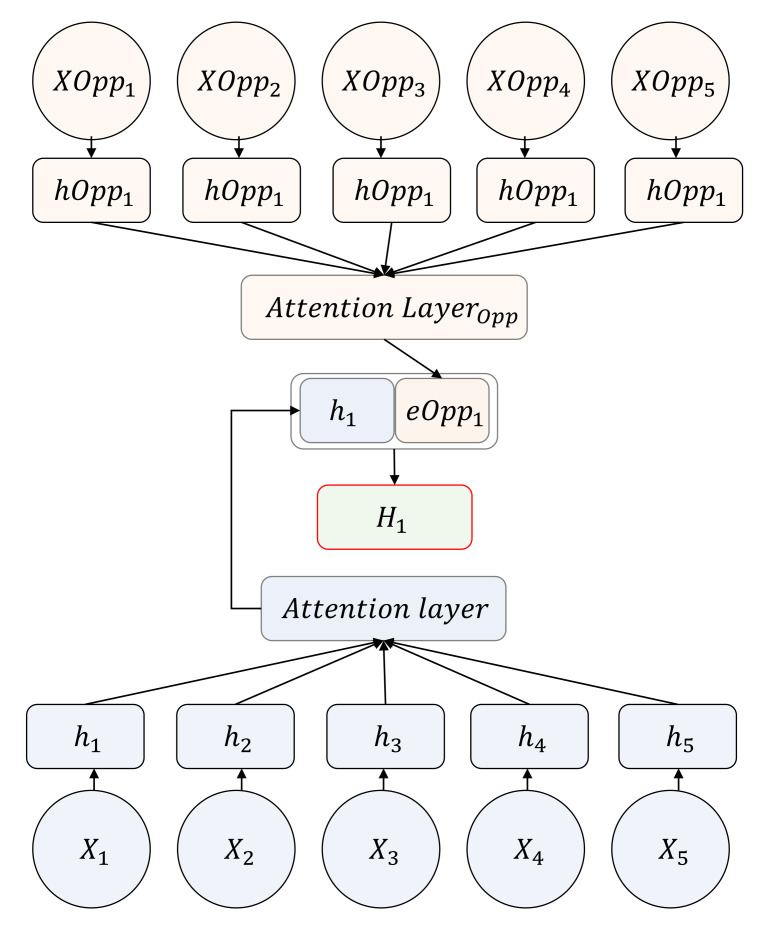
Overall neural network architecture for the multi-agent system: A network architecture expressing 10 multi-agent systems from the perspective of one agent. The feature of five agents in a cooperative relationship is $e_{1}$, while the feature of five agents in a competitive relationship is $h_{1}$. The feature of the entire multi-agent system is $H_{1}$ where $e_{1}$ and $h_{1}$ are concatenated.

Two cooperative–competitive teams with different goals are configured in the same environment. Each multi-agent is represented as a graph consisting of nodes and edges, in which the features for the interaction between agents are effectively extracted through a GNN. Figure 7 shows the overall neural network architecture, where the interaction between five agents in a cooperative relationship and five agents in a competitive relationship among a total of 10 agents is represented from the perspective of one agent.

The set of agents in a cooperative relationship is $S=\{1,2,\cdots ,N_{1}\}$, while the set of agents in a competitive relationship is $S_{Opp}=\left\{N_{1}+1,N_{1}+1,\cdots ,N_{1}+N_{2}\right\}$. The state of each agent is $X_{i}$ and $XOpp_{i}$. $e_{i}$ and $eOpp_{i}$ are the embedded features of multi-agents with a correlation output through the neural network $f_{\theta_{a}}\left(X_{j}\right)$ and $f_{\theta_{b}}\left(XOpp_{j}\right)$. *F* consists of a GNN and attention layer, where the final $H_{i}$ is a value resulting from concatenating $h_{i}$ and $e_{i}$. The process is as follows.

(1) $$e_{i}=f_{\theta_{a}}\left(X_{j}\right),\;\forall j\in S$$

(2) $$eOpp_{i}=f_{\theta_{b}}\left(XOpp_{j}\right),\;\forall j\in S_{Opp}$$

(3) $$H_{i}=concatenate\left(h_{i},e_{i}\right)$$

The policy of a multi-agent system is learned using the final $H_{i}$ and MADDPG. Agents become capable of performing given tasks through MARL by responding to a dynamic environment that changes quickly. Furthermore, the system can respond to an array of circumstances as two groups with different goals are trained together.

### 2.3. Plugin Framework For Neuro-Symbolic

**Figure 8:**
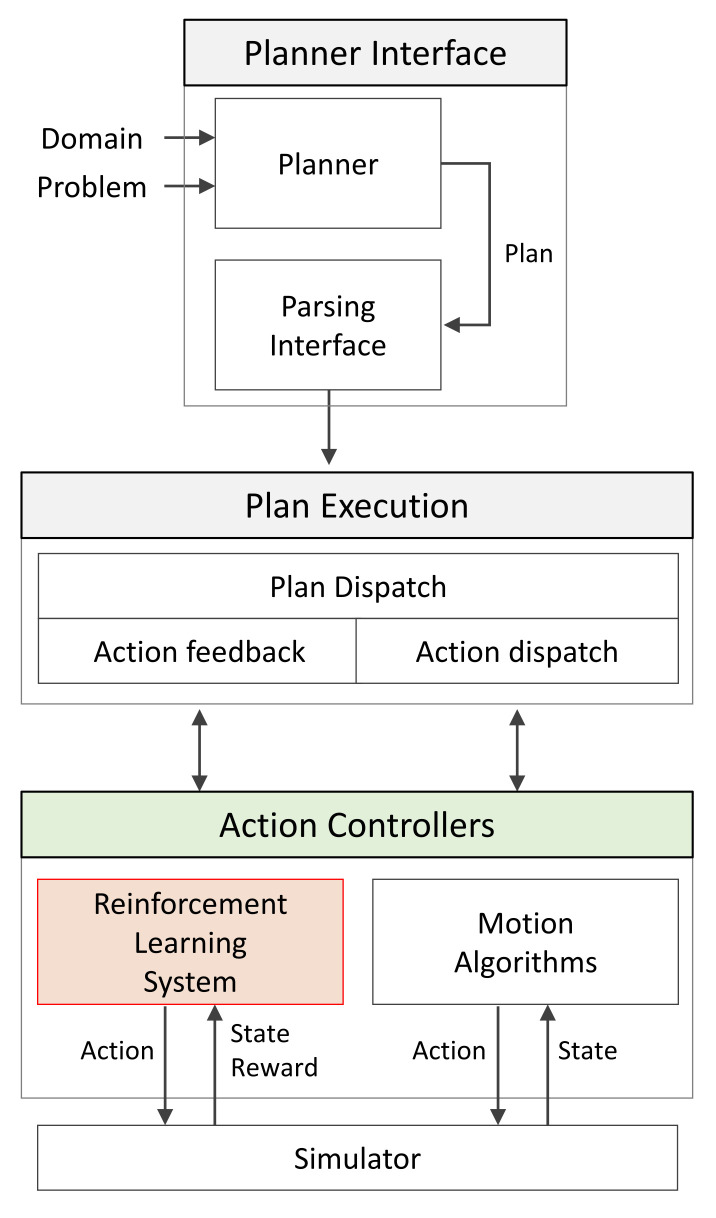
Plugin-based neuro-symbolic framework: Actions generated through a planner are delivered to the action controller through plan execution. Here, agents are removed through the reinforcement learning system if actions requiring a quick response are received. All other actions are applied with motion planning based on motion algorithms.

In this section, a planning system that integrates a hierarchical task planning framework and cooperative–competitive reinforcement learning is discussed. The plugin-based neuro-symbolic framework is as shown in Figure 8. In the planner of the planner interface, a plan is generated by receiving the PDDL domain and problem. A plan signifies the sequence of actions of agents and is delivered to plan execution through a parsing interface. In the planned dispatch, one action that must be performed by each agent of the multi-agent system is sent to the action controller, and all sequences of actions generated through action feedback are delivered. The action controller consists of a reinforcement learning system and motion algorithms and maps the motions that must be performed in a simulator. For motion control in a fast and dynamic environment, corresponding motion algorithms are used in the static environment using the reinforcement learning system. Unlike conventional reinforcement learning methods, our algorithm needs to define the domain in advance. Existing symbolic methods can be used for task planning without learning time, but our method requires time for motion training. However, through the proposed plugin-based neuro-symbolic framework, task planning is solved with a symbolic approach in a wide area and with a connectionist approach in a constantly changing environment.

## 3. Simulation Results

The proposed method is verified in three parts. For symbolic planning, the environment information is updated in real-time using the knowledge base, and it is verified whether the task is executed even when an unexpected situation occurs. For cooperative–competitive reinforcement learning, two teams with different goals are designed, and the experiment is conducted to see whether the task goal is achieved based on Open AI Gym [54] and the configured environment. Lastly, the proposed plugin-based neuro-symbolic framework is verified in a wide and dynamic environment using a multi-agent system consisting of four teams.

### 3.1. Symbolic Planning

**Figure 9:**
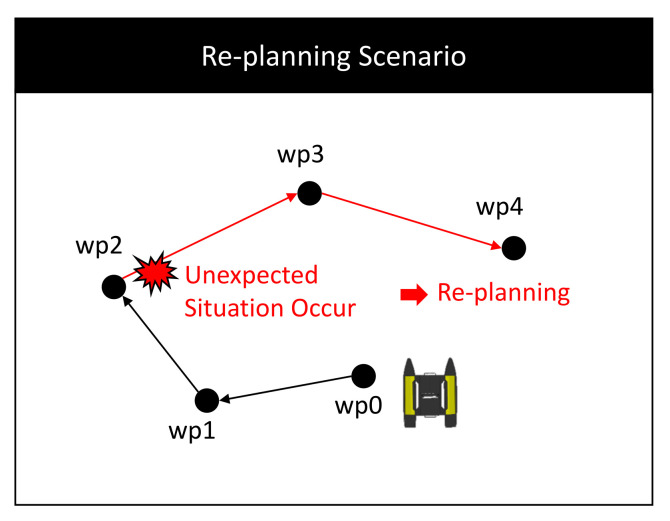
Simulation scenario for re-planning: One USV moves while performing reconnaissance for the given WPs. It was assumed that the mission fails as an unexpected situation occurs at wp2 and it is verified whether re-planning is successfully executed.

Using the knowledge base which stores long-term data and short-term data, the experiment verifies whether the task is successfully re-planned after a failure due to an unexpected situation. The experimental scenario is as shown in Figure 9. One unmanned surface vehicle (USV) surveys a total of six waypoints (WPs). The initial position of the USV is wp0, and the possible action is to move between two WPs. To verify the success of re-planning, it was assumed that an unexpected situation occurred at wp2 during surveillance. The PDDL domain and problem for the surveillance domain were prepared, and the plan of the USV was generated using POPF planner [28]. The results are provided in Appendix A.

The PDDL problem automatically generated using the knowledge base is shown in Table 1. The initial part of Table 1 is the result generated at the beginning of the experiment, while the re-planning part is the result generated again after the task has failed. Since only wp0 was surveilled in the initial part, the init is given the fact (surveilled wp0), while the goal is given the fact regarding other WPs. Since the USV stopped after surveilling wp1 and wp2 during re-planning, the facts (surveilled wp1) and (surveilled wp2) were added to the init, while the surveillance of wp3 and wp4 remained as the goal. Table 2 presents the attributes of the facts stored in the knowledge base. Update type and knowledge type are the attributes for discerning state data and goal data. In this study, MongoDB [55] and the PyMongo library were used to save the data and generate the PDDL problem. The PDDL problem was automatically and successfully generated using the knowledge base.

### 3.2. Cooperative–Competitive Reinforcement Learning

In this section, the multi-agent reinforcement learning method using GNN and MADDPG was verified through two agent-teams with different goals. One team attempted to reach the area of the other team, while the other team attempted to defend and block the opposing team from reaching their area. The experiment was conducted in Open AI Gym, which is most frequently used for verifying reinforcement learning algorithms and in the simulator environment configured in this study. The configured environment and the details of USV are provided in Appendix B. A total of two teams were designed, with each team having different goals and team members cooperating or competing for learning.

**Figure 10:**
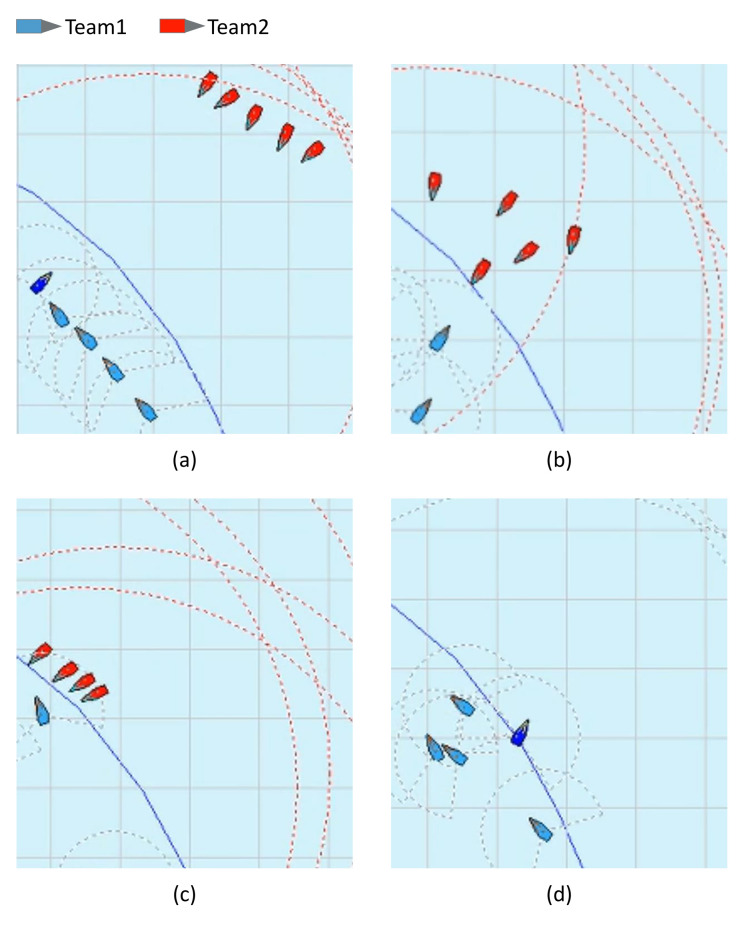
Experimental results of cooperative–competitive reinforcement learning: (**a**) starting positions of the red team and blue team, (**b**) task planning of two teams with different goals, (**c**) red team has reached the blue team’s area, (**d**) blue team has completely defeated red team.

The experimental results of our configured environment are shown in Figure 10. Figure 10a shows the starting points of each team. The goal of the blue team was to surveil and protect their area, while that of the red team was to reach the blue team’s area. In Figure 10c, blue agents were removed and have approached the opponent team’s area. In Figure 10d, red agents were removed and the blue team has protected their area. The experimental results in Open AI Gym are provided in Appendix C. In this study, it was proven through two simulation environments that the multi-agent system successfully cooperated and competed for learning in a rapidly changing dynamic environment.

### 3.3. Plugin Framework for Neuro-Symbolic Approach

**Figure 11:**
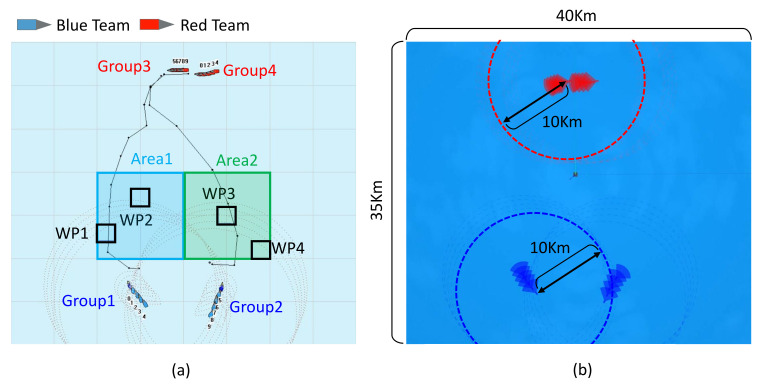
Experimental environment setting to verify the proposed framework: (**a**) executing WP surveillance tasks by operating the blue team and red team consisting of two groups each, (**b**) simulation environment size and LiDAR detection distance of each agent.

The proposed framework was verified in the environment shown in Figure 11. The size of the entire area in which the task was executed is 40 km × 35 km, in which a total of 20 USVs and one COP for controlling all agents were operated. ROS Gazebo based on Ubuntu was used for configuring the task and the simulator for agents, while Windows-based Microsoft Foundation Class (MFC) was used to build the COP system. The blue team and red team each had 20 non-holonomic USVs, where the task was executed in two groups. Each agent was equipped with LiDAR, meaning an object could be detected from up to 10 km. Furthermore, the blue team could eliminate the agents of the red team within 1.5 km. Each agent had a “move” action that allowed movement between points, a “detect” action that allowed the detection of the opponent, and an “eliminate” action that allowed the elimination of the opponent. The task for the blue team was to block the red team, which moved downward while performing surveillance on WPs, while that of the red team was to move to the destination along the given path. The path to the given destination was set in advance, and the red team moved on this path. A surveillance scenario that guarded a specific area while observing the opponent was used to implement the proposed framework.

**Figure 12:**
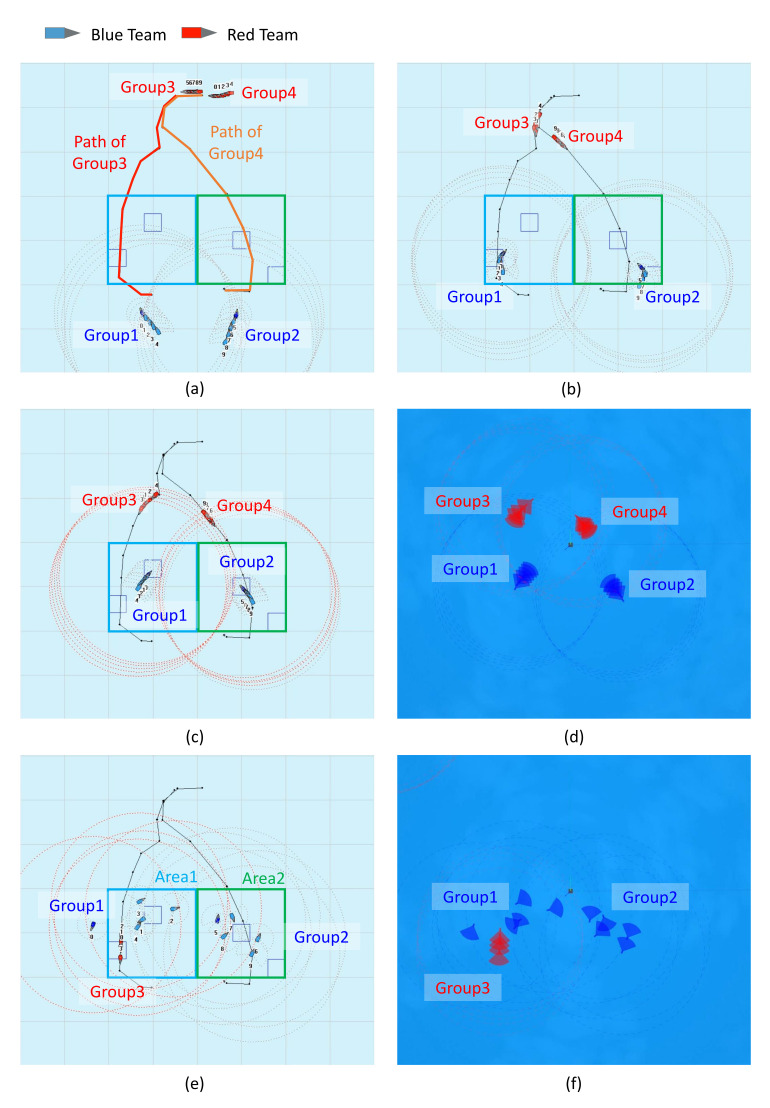
Simulation results of the proposed neuro-symbolic framework: (**a**) initial state, (**b**) blue team performing surveillance in a wide area based on symbolic approach, (**c**) blue team executing surveillance task output from the COP system, (**d**) blue team executing surveillance task in the Gazebo environment, (**e**) group 1 responding with a symbolic approach output from the COP system and group 2 responding to the red team with connectionist approach, (**f**) group 1 responding with symbolic approach in Gazebo environment and group 2 responding to the red team with a connectionist approach.

The proposed method can be applied to other tasks, such as searching, inspecting structures in an extensive environment, and box pushing using the actuator. For instance, the state of the box as the objective can be treated using the symbolic approach and the state of the actuators with the connectionist approach when performing the box pushing operation. However, the action and environments must be defined according to each environment because the proposed method can only be applied to a predefined domain. Therefore, we pre-defined the initial states of working environments and actions. Actions that need to be dealt with rapidly are trained with reinforcement learning in advance. Many instances of task planning can be easily applied to the proposed method if we only verify a motion that should rapidly react to the environment. The evaluation methods and objectives of the symbolic and connectionist approaches are different; therefore, it is difficult to evaluate the proposed method using existing evaluation criteria. This study therefore aims to verify the proposed method through a set of tasks and its results. Groups 3 and 4 of the red team move along the given path as shown in Figure 12a. The blue team performs the surveillance task using the proposed hierarchical task planning and MARL-based framework. For hierarchical task planning, planning at the strategic level is executed in COP while planning at the tactical level is executed by one agent with a planner belonging to each group. The network trained in the cooperative–competitive reinforcement learning section of the simulation results was used in order to configure the reinforcement learning system in Figure 8, which is responsible for responding to a dynamic environment. Figure 12c,d shows the blue team performing the surveillance of given WPs in a wide area based on a symbolic approach. Figure 12e,f shows the blue team counteracting against the red team moving dynamically. It is difficult to respond to the real-time change in environment and agents using the conventional method. Thus, the essential facts for planning were updated in real time to adapt for faster re-planning using the proposed method. Area 1 shows the result of responding using only the conventional symbolic approach, while Area 2 is the result of responding using the proposed framework. The proposed and existing method were both executed in the same environment. The total simulation time was set to 20 min. The time taken for decision making was approximately 1 min for the proposed method and approximately 3 min for the existing method, which required more re-planning owing to environmental changes. Consequently, the blue team could not block the red team in Area 1 but successfully blocked the red team in Area 2. Table 3 shows the comparison result of our approach and the existing method [52]. The existing method was replanned six times, while the proposed method performed the task with only two iterations of replanning. Moreover, our algorithm eliminated the agents of the red team efficiently compared to the conventional method. Hence, the multi-agent system was proven to successfully execute the surveillance task in a wide and dynamic environment using the proposed plugin-based neuro-symbolic architecture.

## 4. Discussion

The core elements of the proposed architecture are the knowledge base and the reinforcement learning system that is plugged in the symbolic planning framework. In the future, we will further study a method for saving the facts in the PDDL-format more efficiently in the knowledge base and a method for applying reasoning to the saved data to infer new information to be utilized in planning. Moreover, other methods for adapting a variety of MARL methods to symbolic approach systems will be researched.

## 5. Conclusions

The size of work environments for agents is continuously increasing and becoming more complex as multi-agent systems are required across diverse fields. Therefore, a task planning framework for a multi-agent system was proposed in this work. For efficient task planning in a wide area, a network-based approach is used that can immediately respond to rapidly changing environments. The proposed plugin-based neuro-symbolic framework consists of hierarchical task planning and cooperative–competitive MARL. A surveillance scenario using one COP and 20 agents in a wide area was prepared, and the experiment was conducted accordingly through a simulation. As a result, it was verified that the multi-agent system successfully executed the task using the proposed method. The proposed method can be applied to various planning tasks (searching, reconnaissance, and structural inspection for various environments, such as ground, underwater, and air). It is particularly suitable for performing dynamic tasks over a wide space; moreover, it is advantageous as predefined or learned actions of agents can be directly applied. However, the proposed method can only be applied to predefined domains; thus, all actions and facts must be defined in advance. Therefore, a method for handling new facts that are not previously defined will be studied in future.

## Figures and Tables

**Table 1:** Automatically generated PDDL problem for initial and re-planning state.

Initial	Re-Planning
(define (problem demo)	(define (problem demo)
(:domain demo)	(:domain demo)
(:objects	(:objects
wp0 wp1 wp2 - waypoint	wp0 wp1 wp2 - waypoint
usv - robot	usv - robot
)	)
(:init	(:init
**(robot****_****at usv wp0)**	**(robot****_****at usv wp2)**
(surveilled wp0)	(surveilled wp0)
)	**(surveilled wp1)**
(:goal (and	**(surveilled wp2)**
** (surveilled wp1)**	)
** (surveilled wp2)**	(:goal (and
(surveilled wp3)	(surveilled wp3)
(surveilled wp4)	(surveilled wp4)
))	))

**Table 2:** Knowledge base data.

Attributes	State Data 1	State Data 2	Goal Data
update type	0	0	1
knowledge type	1	1	1
attribute name	visited	robot at	visited
attribute count	1	2	1
key1	waypoint	robot	waypoint
value1	wp0	usv	wp2
key2	-	waypoint	-
value2	-	wp0	-

**Table 3:** Results of our approach and comparison method.

	Symbolic [52]	Ours
Number of replanning trials	6	2
Blue Team Remaining	5	5
Red Team Remaining	4	0

**Figure A1:**
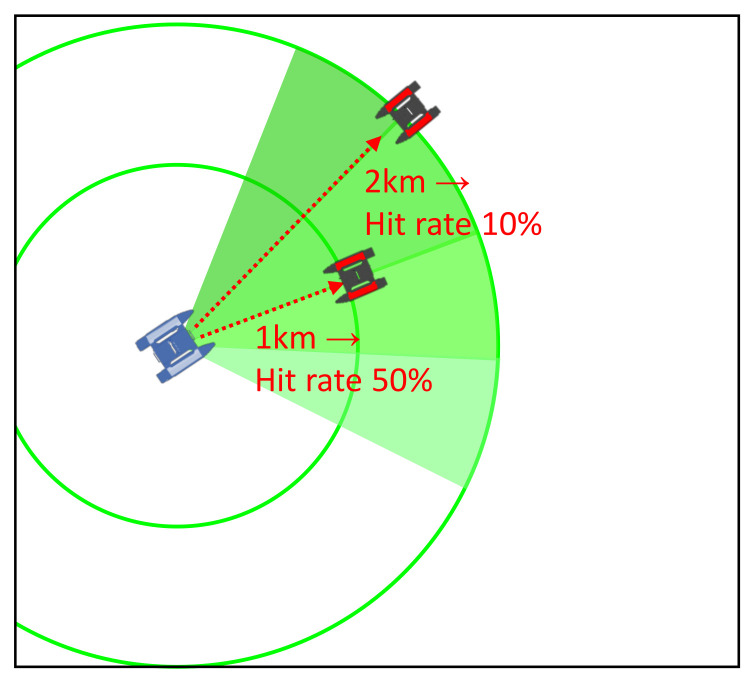
Hit rate of blue agent.

**Figure A2:**
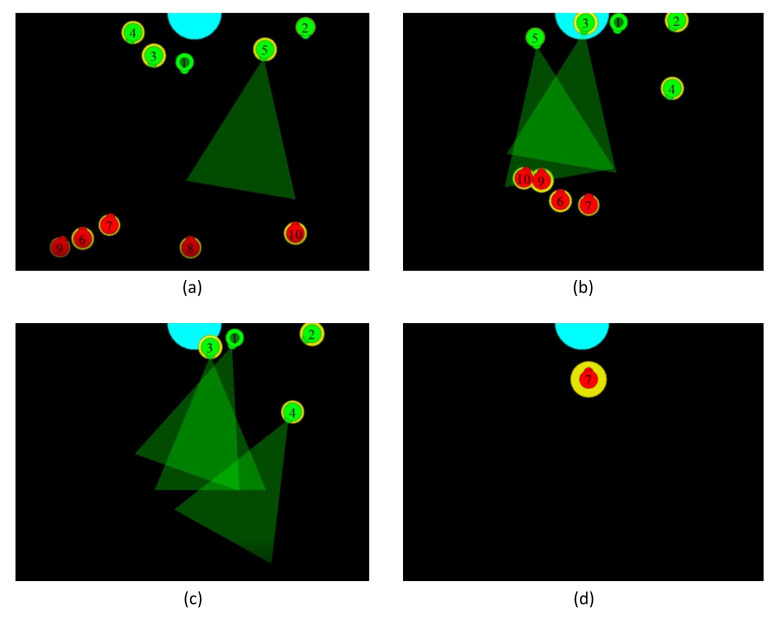
Simulation results of Open AI gym: (**a**) start mission at random point, (**b**) red and green team performed their mission, (**c**) green team defended the fort, (**d**) red team reached the fort.

## Data Availability

Not applicable.

## Abbreviations

The following abbreviations are used in this manuscript:

SLAM	Simultaneous Localization And Mapping
ROMA	Role-oriented multi-agent reinforcement learning
PEORL	Planning execution observation reinforcement learning
PDDL	Planning Domain Definition Language
USV	Unmanned surface vehicles
ROS	Robot operating system
COP	Central operating system
MARL	Multi-agent reinforcement learning
MADDPG	Multi-agent deep deterministic policy gradient
GNN	Graph neural network
WP	Waypoint

## Appendix A

The experimental results output through the PDDL planner are presented in Table A1 and Table A2. The plan generated initially is to perform surveillance by moving from wp1 to wp4, as shown in Table A1. In Table A2, it can be confirmed that the plan generated during re-planning due to an unexpected situation that occurred while performing surveillance only generated the remaining sequence of actions, excluding the actions that were executed previously.

**Table A1:** Initial generated plans.

Initial
(goto_waypoint usv wp0 wp1)
(goto_waypoint usv wp1 wp2)
(goto_waypoint usv wp2 wp3)
(goto_waypoint usv wp3 wp4)

**Table A2:** Generated plans when re-planning.

Re-Planning
(goto_waypoint usv wp2 wp3)
(goto_waypoint usv wp3 wp4)

## Appendix B

The hit rate of the blue agent is as shown in Figure A1. The hit rate at 1 km and at 2 km is 50% and 10%, respectively. The red agents outside the range are not hit.

## Appendix C

The experimental environment, process, and results of Open AI Gym are shown in Figure A2. As shown in Figure A2a, the green team attempts to protect the light blue fort, while the red team attempts to approach the fort. The training results in Figure A2d show that the red team completely eliminated the opposing team and reached the destination, or the green team approached the fort as in Figure A2. The rewards of each team are as shown in Table A3.

**Table A3:** Rewards of green and red team.

Green Team	Red Team
Distance to fort (green agents)	Distance to green team
Distance to fort (red agents)	Distance to fort (red agents)
Fired or not	Fired or not
Red team hit	Green team hit
Red team eliminated	fort reached (yes or no)
